# KPC-producing *Klebsiella pneumoniae* in Switzerland from 2024 to 2025: comparative genomics and cefiderocol resistance mechanisms analyses

**DOI:** 10.1007/s10096-026-05475-8

**Published:** 2026-04-17

**Authors:** Jacqueline Findlay, Patrice Nordmann, NARA network, R Lienhard, L Vonallmen, C Schilt, A Scherler, K Lucke, M Jutzi, M Reichmuth, U Schibli, C Fricker, S Pranghofer, D Blanc, A Vitale, B Lemaire, M Fatoux, M Tritten, L Rumebe, N Liassine, G Jost, N Wohlwend, D Schultze, K Burren, A Westers, M Imperiali, L Pozzi, D Balzari, G Vaninetti, C Cirillo, V Gaia, E Pianezzi, G Martinetti Lucchini, F Baggi Menozzi, A Jayol, C Guyon, D Hyden, M Maitrejean, V Deggi-Messmer, D Bandeira, C Fournier, S Pfister, C Nusbaumer, L Bertaiola Monnerat, J Schrenzel, G Renzi, A Cherkaoui, D Andrey, S Emonet, M Eyer, R Maret, A Belo, D Mabillard, M Moraz, K Herzog, V Gisler, E Hitz, M Oberle, C Castelberg, H Fankhauser, S Graf, N Dubey, C Guler, M Schoenenberger, U Karrer, F Piran, C Andreutti, M Dessauges, T Schmid, B Suterbuser, I Mitrovic, V Bruderer, P Staehli, B Schnell, C O Marti, I Steffen, A Imhof, B Preiswerk, V Dilorenzo, C Payen, D Boschung, L Comte, M Schacher, M Brandenberger, C Zowa, C Zehnder, B Mathis, L Basilico, G Togni, P Minkova, Y Born, M Kuegler, V Povolo, S Droz, M Elzi, C Casanova, C Lang, A Blaich, S Schmid, B Ivan, S Mancini, O Dubois, K Narr, S Schoch, S Ellenberger, S Seiffert, Helena M B Seth-Smith, Adrian Egli, Laurent Poirel

**Affiliations:** 1 Medical and Molecular Microbiology, Faculty of Science and Medicine, University of Fribourg, Fribourg, Switzerland; 2 Swiss National Reference Center for Emerging Antibiotic Resistance (NARA), University of Fribourg, Fribourg, Switzerland; 3 Institute of Medical Microbiology, University of Zürich, Zürich, Switzerland

**Keywords:** *Klebsiella pneumoniae*, Carbapenemase, KPC, Cefiderocol, Plasmid, *Fec* operon

## Abstract

**Objectives:**

KPC-producing *Klebsiella pneumoniae* (KPC-KP) are increasingly prevalent in Europe, largely due to the dissemination of a combination of successful plasmids and clones. The aim of this study was to characterise, both phenotypically and genotypically - with a focus on cefiderocol resistance - the KPC-KP isolates submitted to the Swiss National Reference Centre for Emerging Antibiotic Resistance (NARA), from February 2024 to February 2025.

**Methods:**

Sixty-six non-duplicate KPC-KP isolates were submitted to NARA over the study period from across Switzerland. Antimicrobial susceptibility was determined using a combination of disk testing, E-tests and broth microdilution, and interpreted according to EUCAST methodology. Whole-genome sequencing (WGS) was performed on all isolates to allow for further characterisation. WGS analyses and assemblies were performed using the IMMense pipeline. KPC variants were cloned into vector pUCP24 and transformed into *Escherichia coli* Top10.

**Results:**

The most prevalent KPC variants identified in this study were KPC-3 (*n* = 49), KPC-2 (*n* = 13), KPC-184 (*n* = 2), KPC-39 (*n* = 1), and one isolate harboured a novel KPC allele, designated KPC-272. Within the 66 isolates, 24 sequence types (STs) were ascertained, represented by one to three isolates with the exceptions of ST307 (*n* = 14), ST512 (*n* = 8), and ST258 (*n* = 7), ST39 (*n* = 5) and ST101 (*n* = 5). All isolates exhibited resistance to cephalosporins and carbapenems and, notably, 18 isolates (27.3%) were cefiderocol (FDC) -resistant. Analysis of WGS data for potential mechanisms of FDC resistance identified that 15/18 isolates harboured a plasmid-mediated *fec* operon, and 4/18 isolates carried expanded-spectrum beta-lactamases that have been reported to contribute to FDC resistance.

**Conclusions:**

This study determined that the current dissemination of KPC-KP isolates in Switzerland is polyclonal and not related to a single predominant and “successful” clone. The elevated rate of resistance to the “last resort” antimicrobial, FDC, is concerning, and was shown to be caused by multifactorial mechanisms.

**Supplementary Information:**

The online version contains supplementary material available at https://doi.org/10.1007/s10096-026-05475-8.

## Introduction

The increased incidence of infections caused by carbapenem-resistant *Klebsiella pneumoniae* (CRKP) globally, particularly when associated with healthcare-associated infections, constitutes a major cause of concern for public health [1, 2]. CRKP are often multidrug-resistant, accumulating additional resistance mechanisms and leaving few available treatment options. This has led to their classification by the World Health Organisation as a critical priority pathogen in its Bacterial Priority Pathogens List [3]. *K. pneumoniae* is the third most common cause of bacteriaemia after *Escherichia coli* and *Staphylococcus aureus*, and infections caused by CRKP result in a significantly higher mortality rate relative to their sensitive counterpart [4].

Within Europe, *Klebsiella pneumoniae* carbapenemase (KPC)-type enzymes are the most common carbapenemases reported in *K. pneumoniae*, accounting for 46% of all carbapenemases [5]. KPC-encoding genes are typically located within mobile genetic elements, most often the ~ 10-kb Tn*4401* transposon, that usually disseminate via plasmids [6]. The plasmid pKpQIL was first described in a *K. pneumoniae* ST258 isolate from Israel in 2010 [7], and pKpQIL-like plasmids have since gone on to become the most successful vehicles for the spread of *bla*_KPC_ within *K. pneumoniae* and amongst other Enterobacterales species globally [6]. However, numerous plasmids of various replicon types have since been associated with the spread of KPC encoding genes, notably IncX3. This plasmid type is particularly prevalent in East Asia, and is an example of how the dominance of particular plasmids can vary geographically [5]. Successful plasmids in combination with the emergence and the dissemination of high-risk clones, including members of clonal group 258 (ST258, ST512, ST11) and ST307, have been pivotal to the success of KPC-producing *K. pneumoniae* (KPC-KP) [1, 5].

In recent years several novel antibiotics including cefiderocol (FDC) and beta-lactam-beta-lactamase inhibitor (BLBLI) combinations such as ceftazidime-avibactam (CZA), imipenem-relebactam (IPR), and meropenem-vaborbactam (MEV) have been approved for the treatment of infections caused by KPC-KP. However, reports of the emergence of resistance to these antimicrobials have been quick to materialise following their approval for use. Resistance is often attributed to a combination of mechanisms including KPC mutations (particularly for CZA and FDC), porin mutations, and *bla*_KPC_ gene amplification [8–10]. In recent years, there have been numerous reports of KPC variants emerging, often as a direct consequence of antibiotic selective pressure with CZA, that are capable of conferring resistance to both and CZA and FDC [9, 11, 12]. Both CZA and FDC are considered to be critical/”last resort” antimicrobials for the treatment of CRKP, and as such the emergence of resistance to these treatments is particularly worrisome. Therefore tracking the epidemiology of KPC-KP to recognise phenotypic and genotypic patterns of FDC/CZA-resistant isolates, to countermeasure CRKP has become crucial. In this context, this study aimed to describe and characterise contemporary KPC-KP isolates received at the Swiss National Reference Centre for Emerging Antibiotic Resistance (NARA) over a 12-month period, from February 2024 to February 2025.

## Materials and methods

### Bacterial isolates and identification

Over a 12-month period, from February 2024 – February 2025, NARA received a total of 76 KPC-positive *K. pneumoniae* isolates from hospitals and clinics throughout Switzerland. In Switzerland, it is mandatory for all carbapenem-resistant Enterobacterales to be sent to the NARA. Identification at the species level was initially performed using API20E (bioMérieux) and then further assessed through the analysis of the whole genome sequence data. All isolates were subjected to the Rapidec Carba NP test (bioMérieux) and then to NG-Test CARBA 5 test (NG Biotech), according to the manufacturer’s instructions. Isolates that tested positive by the Rapidec Carba NP test and the NG-Test CARBA 5 test were screened by PCR for the presence of the KPC alleles. Patient and isolation source data was obtained from the accompanying request forms sent by referring laboratories.

### Susceptibility testing

Antimicrobial susceptibility testing was performed by disk diffusion, broth microdilution (BMD), or by E-test in the cases of CZA, IPR, and MEV. MICs of FDC were determined by BMD using iron-depleted Mueller-Hinton broth. For MICs of FDC + avibactam (AVI), AVI was used at a fixed concentration of 4 mg/L. All susceptibility testing was performed and interpreted following EUCAST guidelines (version v15.0) [13].

### Cloning of KPC alleles

The *bla*_KPC_ alleles encoding KPC-2, KPC-3, KPC-39, KPC-184 and KPC-272, were amplified by PCR and cloned into vector pUCP24 [14] using primers KPC-SacIF (5′-GATGATGAGCTCCTGATTACATCCGGCCGCTA-3′) and KPC-BamHIR (5′-GATGATGGATCCCGGCGGTGGTGGGCCAATA -3′), before transformation into *E. coli* Top10. Transformants were selected on plates supplemented with 25 mg/L gentamicin. Successful transformants were confirmed by amplification and sequencing of the KPC alleles.

### Whole-genome sequencing and analyses

Whole-genome sequencing (WGS) was performed on 66 nonduplicate isolates (by patient, KPC variant and phenotype) on a NextSeq 1000 instrument (Illumina, https://www.illumina.com) with 2 × 150 bp paired end reads following QiaSeq FX (Qiagen) library preparation. Bioinformatic analyses for Quality Control, including minimum mean read depth of 30x and contamination checks, and assemblies, were performed using IMMense, an in-house developed pipeline based on Nextflow (https://gitlab.uzh.ch/appliedmicrobiologyresearch/immense), as previously described [15]. Genome assemblies were typed in Ridom Seqsphere+ v10.5.4 by multi-locus sequence typing (MLST). Speciation was confirmed using KmerFinder 3.2 and plasmid replicon types were identified PlasmidFinder 2.1,16 and KmerFinder 3.2 on the Center for Genomic Epidemiology platform (https://cge.cbs.dtu.dk/services). Capsular and O antigen types, and virulence scores were determined using Kleborate 3.2.4 (https://github.com/klebgenomics/Kleborate) [16]. A core genome single-nucleotide polymorphism (SNP) alignment [17] was generated using Pathogenwatch (https://pathogen.watch) and viewed using Interactive Tree of Life version 7.4.2 [18].

The plasmid sequence of pKpQIL (GenBank Accession No. NC014016) was downloaded from GenBank and used as a reference for read mapping using the Geneious mapper (https://www.geneious.com/) on high sensitivity settings with 5 iterations. Assembled contigs of the complete genomes were then mapped using progressive Mauve alignment software to manually mitigate against false positives. A > 90% coverage and identity were used to assess relevant matches.

Sequence data from this study was submitted to the National Center for Biotechnology Information’s Sequence Read Archive (BioProject no. PRJNA1423521).

## Results and discussion

### Isolate sources and demographics

**Fig. 1:**
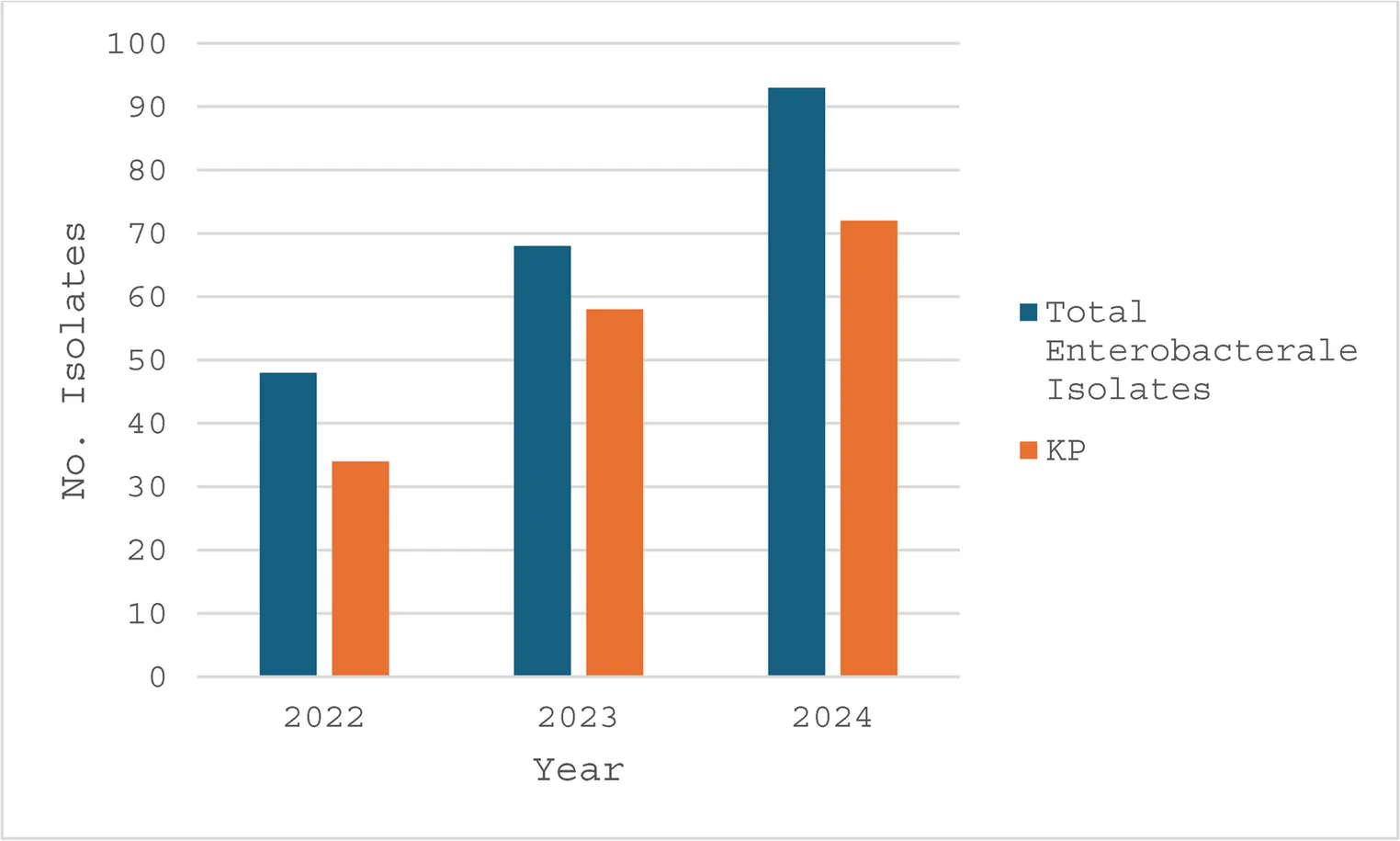
Numbers of KPC-positive Enterobacterales and KPC-positive *K. pneumoniae* isolates received by NARA from 2022–2024. KP; *K. pneumoniae*

Over the previous three years, from 2022 to 2024, the numbers of KPC-positive Enterobacterales (KPC-E) isolates submitted to NARA have steadily increased, the vast majority of which (71–85%) were *K. pneumoniae* (Fig. 1). Over a 12-month period, between Feb 2024 to Feb 2025, NARA received a total of 98 KPC-E, of which 76 were KPC-KP, and following deduplication (by patient, KPC variant and phenotype), a total of 66 isolates remained for analysis. Over half of those isolates (35/66; 53.0%) were obtained from screening swab samples (including faecal and rectal) and the remaining isolates were from urine (17/66; 25.8%), tissue and fluid (5/66; 7.6%), or respiratory samples (5/66; 7.6%), and the remaining 4 isolates were from blood cultures (4/66; 6%). Almost two-thirds of isolates were obtained from males (43/66; 65.2%). Isolates were sent from laboratories across 12 Swiss Cantons and from all seven major regions of Switzerland, with most isolates being sent from the Lake Geneva region (18/66; 27.3%) and Zurich region (18/66; 27.3%), which is in line with the population density of these regions.

### Typing, KPC alleles and Tn*4401* isoforms

**Fig. 2:**
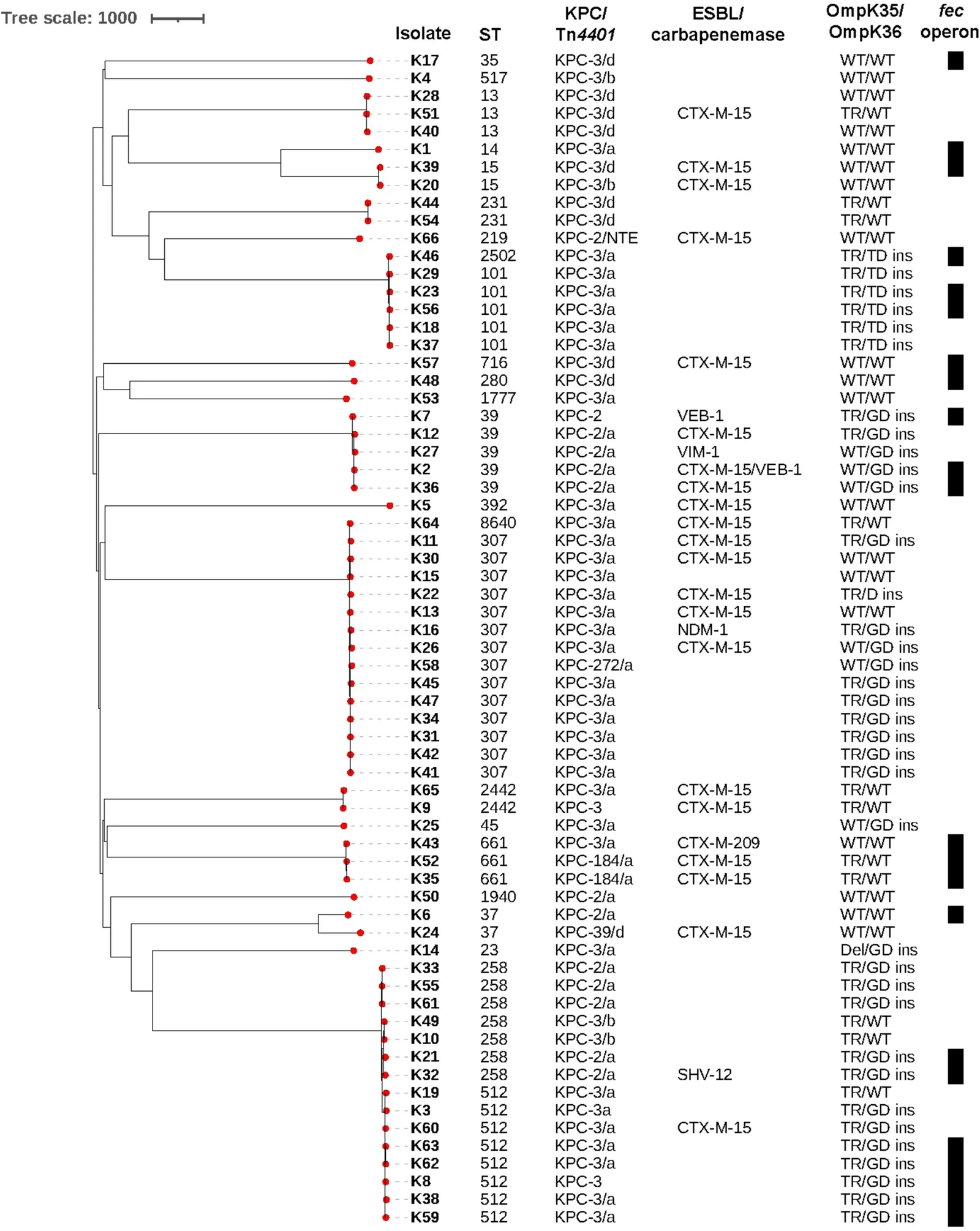
Core genome snp tree of all 66 KPC-KP isolates. TR; truncated, WT; wild-type, GD ins; GD insertion in L3 loop, TD ins; TD insertion in L3 loop, D ins; D insertion in L3 loop, Del; gene deleted

WGS confirmed that all 66 isolates were *K. pneumoniae* KpI and within these, 24 sequence types (STs) were ascertained. Most STs were represented by no more than 3 isolates with the exceptions of ST307 (*n* = 14), ST512 (*n* = 8), and ST258 (*n* = 7) (the two latter STs being members of clonal group (CG)-258). In addition, ST39 (*n* = 5) and ST101 (*n* = 5) were found. Members of CG-258 have been associated with the spread of *bla*_KPC_ globally, with members of this CG exhibiting success in different regions. For example, KPC-3-producing ST512 (a single locus variant (SLV) of ST258) has become endemic in several countries but most notably in Italy [19], and KPC-2-producing ST11 (another SLV of ST258) is the predominant clone in China [1]. Five KPC alleles were identified; KPC-3 (*n* = 49), KPC-2 (*n* = 13), KPC-184 (*n* = 2), KPC-39 (*n* = 1), and one isolate harboured a novel KPC allele. This novel KPC allele was later designated KPC-272 (GenBank Accession No. PV615266). KPC-2 and KPC-3 are the most common variants globally but to date, a total of 279 variants have been described (http://www.bldb.eu/). This number continues to increase steadily, especially the CZA and FDC-resistant variants (Fig. 2).

Typically, *bla*_KPC_ is encoded within a Tn*3*-based transposon, Tn*4401*, of which there are currently 13 reported isoforms (labelled *a* to *m*). The isoforms differ by varying insertion or deletions within a polymorphic region upstream of the *bla*_KPC_ allele, affecting its promoter, and resulting in different levels of *bla*_KPC_ expression [20, 21]. Globally, isoform Tn*4401a* is the most prevalent followed by Tn*4401b*. However, Tn*4401a* has been reported to result in significantly higher level of *bla*_KPC_ expression (> 2-fold higher) relative to Tn*4401b* [20]. Within the 66 isolates in this study, three isoforms were detected; Tn*4401a* (51/66; 77.3%), Tn*4401b* (4/66; 6.1%), Tn*4401d* (10/66; 15.2%), and one isolate harboured the *bla*_KPC_ gene within a non-Tn*4401* element (NTE).

### Antibiotic susceptibility

As could be expected for KPC-producing *K. pneumoniae*, all isolates were non-susceptible to most tested beta-lactams (Table S1), and additionally four isolates exhibited resistance to CZA. Two of these isolates co-produced an MBL (NDM-1 or VIM-1), and the remaining two isolates harboured either *bla*_KPC−184_ or a novel KPC variant, subsequently designated *bla*_KPC−272_ (GenBank Accession No. PV615266). KPC-184 differs from KPC-3 by a single amino acid change at position 277 (E277D) within the 270-loop, whilst KPC-272 harbours an EL duplication within the enzyme omega loop and additionally a five amino acid insertion (NSEAV) within the 270-loop. Interestingly, one isolate, K24, that harboured *bla*_KPC−39_ remained sensitive to CZA despite the fact that this variant had been reported as a source of resistance to CZA [22]. It should be acknowledged that the reported KPC-39 *K. pneumoniae* exhibited a moderate MIC of CZA (12 mg/L), and the MIC of the corresponding *E. coli* recombinant strain was only 4 mg/l [22]. Considering that no porin deficiencies could be identified through WGS analyses, this low MIC of CZA for isolate K24 is therefore not so surprising. Susceptibility testing of recombinant *E. coli* Top10 strains producing each of the five KPC variants found in this study showed that variants KPC-39 and KPC-272 conferred elevated MICs to CZA and FDC, whilst corresponding *E. coli* producers remaining susceptible to the carbapenems, relative to KPC-2/KPC-3 (Table 1). However, KPC-184 conferred a phenotype similar to KPC-2/KPC-3, suggesting that the CZA-resistant phenotype observed for isolate K35 was due to another mechanism. Of note, the “trade-off” of decreased susceptibility to CZA and increased susceptibility to the carbapenems, is frequently observed in KPC variants with mutations or indels in the omega loop [23]. Just two isolates exhibited resistance to the BLBLI combinations, namely MEV and IPR, both of which produced either NDM-1 or VIM-1 metallo-ß-lactamases (MBLs), which are not inhibited by relebactam (REL) or vaborbactam (VAB). Interestingly, 18/66 isolates (27.3%) exhibited MICs to FDC > 2 mg/L (ranging from 4 to 64 mg/L), deeming them resistant according to EUCAST guidelines (Table 2) [13]. FDC resistance was observed across 8 STs, indicating that no single lineage was responsible of such phenotypic trait on its own. Eight of the FDC-R isolates were obtained from clinical infection samples whilst the remaining 10 were from screening swabs.

**Table 1:** MICs of recombinant strains expressing the different KPC variants found in this study

Strain	MIC (mg/L)				
	CAZ	CZA	IPM	MEM	FDC
EC Top10	≤ 0.25	≤ 0.25	≤ 0.25	≤ 0.25	≤ 0.06
EC Top10/pUCP-KPC-2	8	≤ 0.25	32	8	0.125
EC Top10/pUCP-KPC-3	64	≤ 0.25	32	8	0.125
EC Top10/pUCP-KPC-39	16	1	≤ 0.25	≤ 0.25	0.5
EC Top10/pUCP-KPC-184	64	≤ 0.25	64	16	0.25
EC Top10/pUCP-KPC-272	16	8	≤ 0.25	≤ 0.25	0.5

CAZ; ceftazidime, CZA; ceftazidime-avibactam, IPM; imipenem, MEM; meropenem, FDC; cefiderocol.

**Table 2:** Phenotypic and genotypic characteristics of 18 FDC-resistant isolates

Isolate		MIC (mg/L)		OMPs		Carbapenemases/ESBLs				
	ST	FDC	FDC-AVI	OmpK35	OmpK36	KPC Variant/Tn4401 isoform	Additional Carbapenemases/ESBLs	Known contributory FDC resistance mechanisms	Virulence Score	K type/O type
K2	39	8	1	FL	GD ins	KPC-2/a	CTX-M-15, VEB-1	*fec* operon, VEB-1	1	KL23/O1/O2v2
K8	512	4	2	TR	GD ins	KPC-3/a		*fec* operon	0	KL107/O1/O2v2
K10	258	8	1	TR	FL	KPC-3/b			0	KL25/O5
K16	307	64	32	TR	GD ins	KPC-3/a	NDM-1	EnvZ Val145Gly, NDM-1	0	KL102/O1/O2v2
K23	101	8	1	TR	TD ins	KPC-3/a		*fec* operon	4	KL17/O1/O2v1
K24	37	16	1	FL	FL	KPC-39/d	CTX-M-15	KPC-39	0	KL118/OL101
K32	258	8	0.5	TR	GD ins	KPC-2/a	SHV-12	*fec* operon, SHV-12	1	KL106/O1/O2v2
K35	661	8	4	TR	FL	KPC-184/a	CTX-M-15, SHV-27	*fec* operon	1	KL27/O1/O2v2
K36	39	4	0.5	FL	GD ins	KPC-2/a	CTX-M-15	*fec* operon	0	KL23/O1/O2v2
K38	512	4	2	TR	GD ins	KPC-3/a	SHV-27	*fec* operon	4	KL107/O1/O2v2
K39	15	4	0.5	FL	FL	KPC-3/d	CTX-M-15	*fec* operon	1	KL19/O1/O2v2
K43	661	4	0.5	FL	FL	KPC-3/a	CTX-M-209	*fec* operon	0	KL27/O1/O2v2
K46	2502	4	2	TR	GD ins	KPC-3/a		*fec* operon	1	KL17/O1/O2v1
K52	661	8	4	TR	FL	KPC-184/a	CTX-M-15, SHV-27	*fec* operon	1	KL27/O1/O2v2
K56	101	4	2	TR	TD ins	KPC-3/a		*fec* operon	0	KL17/O1/O2v1
K59	512	4	2	TR	GD ins	KPC-3/a		*fec* operon	1	KL107/O1/O2v2
K62	512	4	2	TR	GD ins	KPC-3/a		*fec* operon	1	KL107/O1/O2v2
K63	512	4	2	TR	GD ins	KPC-3/a			1	KL107/O1/O2v2

FL; full length, TR; truncated, GD ins; GD insertion in L3 loop, TD ins; TD insertion in L3 loop.

### Analysis of porins, OmpK35 and OmpK36

Sequence analysis of the genes encoding the outer membrane porin OmpK35 identified that the porin was non-functional due to either a disruption resulting in a premature stop codon or deleted in 41/66 (62.1%) isolates. OmpK36 was wildtype in 30/66 (45.5%) isolates and the remaining 36 isolates had insertions within the L3 loop; GD (*n* = 29; comprising ST307 (*n* = 10), ST512 (*n* = 7), ST39 (*n* = 5), ST258 (*n* = 5), ST23 (*n* = 1), ST45 (*n* = 1)), TD (*n* = 6; all ST101 or ST101 SLVs), or D (*n* = 1; ST307). Insertions within the OmpK36 L3 loop, including GD, TD, and D insertions, have been shown to result in a constriction of the OmpK36 porin that can severely hinder the entrance of beta-lactams into the cell, particularly the carbapenems, and result in elevated MICs [24].

### KPC plasmids

Analysis of plasmid replicon types identified that 32 isolates harboured both the FIIK and FIBpQIL replicons types that are characteristic of the pKpQIL plasmid, one of the most commonly reported vehicles of dissemination of KPC encoding genes [6, 7]. Mapping of the sequencing reads against the pKpQIL plasmids identified that 29 isolates exhibited > 90% coverage and > 97% identity, indicating a strong likelihood that the isolates harbour a pKpQIL-like plasmid. However, one limitation of using short-read sequencing data for mapping analysis and in the absence of long-read sequencing data, these analyses can only be considered indicative and not definitive.

### Virulence

Kleborate was used to define the surface polysaccharide loci (capsule and O antigen) and the virulence profiles/scores of the 66 KPC-KP isolates (Table 2 and Table S2). According to the Kleborate virulence score interpretation, four isolates cold be considered hypervirulent with virulence scores of 4 (aerobactin and yersiniabactin positive), two of which were also FDC-resistant (Table 2).

### Cefiderocol resistance mechanisms

#### The *fec* operon

To assess the possible mechanisms of FDC-resistance, all 66 genomes were analysed for the presence of a plasmid-mediated *fec* operon. This operon has previously been shown to result in elevated MICs to FDC [25, 26] and can be considered significant contributor towards FDC resistance. A recent study assessed the prevalence of the *fec* operon in 27,793 *K. pneumoniae* whole genome sequences and found that it was present in 38.4% of genomes [26]. Amongst the 66 isolates in this study, 22 (33%) harboured the *fec* operon, and 15 of these 22 isolates (68%) exhibited resistance to FDC, with MICs ranging between 4 and 16 mg/L, and the remaining 7/22 (32%) isolates exhibited MICs ranging between 0.5 and 2 mg/L. Overall, only three out of the 18 FDC-resistant isolates did not harbour a plasmid-mediated *fec* operon (Table 2). The *fec* operon identified from the isolates of this study was identical to that described in previous reports [25, 26].

#### Beta-lactamase variants

Within the 18 FDC-resistant strains, the addition of 4 mg/L avibactam resulted in significant reductions (≥ 4-fold) in the MICs in 7 isolates, suggesting that the resistance was conferred by an enzymatic mechanism. Two isolates harboured genes encoding KPC variants conferring reduced susceptibility to CZA and FDC (KPC-39 and KPC-272; Table 1), one harboured the ESBL gene *bla*_SHV−12_, and one the ESBL gene *bla*_VEB−1_. All four enzymes have been reported in this study or in previous studies to confer at least reduced susceptibility to CZA and FDC, and so are likely responsible for the elevated FDC MICs observed in these isolates [11, 27, 28]. It could be hypothesised in these cases that the FDC resistance may have been inadvertently selected for by the use of CZA rather than by the use of FDC itself, considering that use of FDC still remains exceptional in the country.

#### Chromosomal mutations

Chromosomal mutations in *fiu* and *cirA*, both encoding TonB-dependent iron transporters, have been shown to result in decreased susceptibility to FDC [29]. Additionally, mutations within *envZ*, a gene encoding a sensor histidine kinase that forms part of the EnvZ/OmpR two-component system, have also been shown to result in elevated FDC MICs [12, 30]. Within the 66 isolates in this study, no mutations were detected in either *fiu* or *cirA*, but one isolate, namely K16, exhibiting a high MIC of FDC (64 mg/L) and producing NDM-1, harboured an amino acid substitution (Val145Gly) in EnvZ. A previous publication showed that a change in the same regions of the protein, Val147Gly, was sufficient to elevate both FDC and CZA MICs significantly [12].

## Conclusions

This study that analysed the contemporary KPC-producing *K. pneumoniae* isolates recovered from patients across Switzerland generated an updated figure about the distribution and phylogeny of those problematic bacteria. Of note, KPC-KP represented ca. 18% of the carbapenemase-producing *K. pneumoniae* received by NARA, the remaining isolates comprising OXA-48-like producers (43%), NDM-like producers (36%), and VIM-like producers (3%). This study illustrated that the current dissemination of KPC-KP isolates was polyclonal and not related to a single predominant and “successful” clone. The epidemiological features observed are similar to that reported in neighbouring countries, with similar strain backgrounds circulating [1]. The variety of FDC resistance mechanisms identified in this study emphasise that resistance is often complex and multifactorial, requiring high-resolution methods, including WGS, to adequately monitor. The elevated rate of resistance to FDC, a “last resort” antimicrobial, is concerning and underlies the necessity for the continued monitoring of FDC-resistant isolates, in combination with antimicrobial stewardship, to preserve the efficacy of this antimicrobial.

## Supplementary Information

Below is the link to the electronic supplementary material.

Supplementary Material 1

## Data Availability

No datasets were generated or analysed during the current study.
