# Supplementary material for: KPC-producing Klebsiella pneumoniae in Switzerland from 2024 to 2025: comparative genomics and cefiderocol resistance mechanisms analyses
Source: Eur J Clin Microbiol Infect Dis. 2026 Apr 17;45(8):2335–43. doi: 10.1007/s10096-026-05475-8 (PMC13652887; doi:10.1007/s10096-026-05475-8)
Supplement: Supplementary file 1 [file 10096_2026_5475_MOESM1_ESM.docx]

|  | **Susceptibility (S/I/R)** | | | | | | | | | | | | | | | | | | |
| --- | --- | --- | --- | --- | --- | --- | --- | --- | --- | --- | --- | --- | --- | --- | --- | --- | --- | --- | --- |
| **Isolate** | **AMX** | **AMC** | **PIP** | **PTZ** | **TEM** | **FOX** | **CTX** | **CAZ** | **CTX** | **FEP** | **ATM** | **ETP** | **IPM** | **MEM** | **CZA** | **FDC** | **FDCA** | **IPR** | **MEV** |
| K1 | R | R | R | R | R | R | R | R | R | R | R | R | I | I | S | 1 | 1 | 0.019 | 0.023 |
| K2 | R | R | R | R | R | R | R | R | R | R | R | R | R | R | S | 8 | 1 | 0.38 | 0.38 |
| K3 | R | R | R | R | R | R | R | R | R | R | R | R | R | R | S | 0.5 | 0.5 | 0.25 | 1 |
| K4 | R | R | R | R | I | I | R | R | R | I | R | R | S | I | S | 0.25 | ≤0.06 | 0.125 | 0.03 |
| K5 | R | R | R | R | R | R | R | R | R | R | R | R | R | R | S | 1 | 0.125 | 0.25 | 0.047 |
| K6 | R | R | R | R | R | R | R | R | R | R | R | R | R | R | S | 0.5 | 0.125 | 0.38 | 0.094 |
| K7 | R | R | R | R | R | R | R | R | R | R | R | R | R | R | S | 0.5 | 0.125 | 0.38 | 1.5 |
| K8 | R | R | R | R | R | R | R | R | R | R | R | R | R | R | S | 4 | 2 | 0.5 | 2 |
| K9 | R | R | R | R | R | R | R | R | R | R | R | R | R | R | S | 1 | ≤0.06 | 0.25 | 0.032 |
| K10 | R | R | R | R | R | R | R | R | R | R | R | R | R | R | S | 8 | 1 | 1.5 | 1.5 |
| K11 | R | R | R | R | R | R | R | R | R | R | R | R | R | R | S | 0.5 | ≤0.06 | 0.5 | 3 |
| K12 | R | R | R | R | R | R | R | R | R | R | R | R | R | R | S | 1 | ≤0.06 | 0.5 | 1.5 |
| K13 | R | R | R | R | R | R | R | R | R | R | R | R | R | R | S | 0.5 | ≤0.06 | 0.19 | 0.064 |
| K14 | R | R | R | R | R | R | R | R | R | R | R | R | R | R | S | 0.5 | ≤0.06 | 0.002 | 0.125 |
| K15 | R | R | R | R | R | R | R | R | R | R | R | R | R | R | S | 0.25 | ≤0.06 | 0.25 | 0.25 |
| K16 | R | R | R | R | R | R | R | R | R | R | R | R | R | R | R | 64 | 32 | >32 | >64 |
| K17 | R | R | R | R | I | I | R | R | R | R | R | R | I | I | S | 2 | 0.5 | 0.25 | 0.047 |
| K18 | R | R | R | R | R | R | R | R | R | R | R | R | R | R | S | 0.5 | 0.125 | 0.38 | 2 |
| K19 | R | R | R | R | R | R | R | R | R | R | R | R | R | R | S | 0.5 | ≤0.06 | 0.19 | 0.023 |
| K20 | R | R | R | R | I | I | R | R | R | R | R | R | I | I | S | 0.5 | ≤0.06 | 0.25 | 0.047 |
| K21 | R | R | R | R | R | R | R | R | R | R | R | R | R | R | S | 4 | 1 | 1 | 3 |
| K22 | R | R | R | R | R | R | R | R | R | R | R | R | R | R | S | 1 | ≤0.06 | 0.75 | 8 |
| K23 | R | R | R | R | R | R | R | R | R | R | R | R | R | R | S | 8 | 1 | 0.25 | 0.5 |
| K24 | R | R | R | R | I | I | R | R | R | R | R | S | R | S | S | 16 | 1 | 0.25 | 0.032 |
| K25 | R | R | R | R | R | R | R | R | R | R | R | R | R | R | S | 0.5 | ≤0.06 | 0.38 | 0.125 |
| K26 | R | R | R | R | R | R | R | R | R | R | R | R | R | R | S | 0.5 | ≤0.06 | 0.75 | 1 |
| K27 | R | R | R | R | R | R | R | R | R | R | R | R | R | R | R | 0.5 | 0.125 | >32 | >64 |
| K28 | R | R | R | R | R | R | R | R | R | R | R | R | R | I | S | 0.25 | 0.125 | 0.25 | 0.047 |
| K29 | R | R | R | R | R | R | R | R | R | R | R | R | R | R | S | 0.5 | ≤0.06 | 0.5 | 1 |
| K30 | R | R | R | R | R | R | R | R | R | R | R | R | S | I | S | 0.5 | ≤0.06 | 0.032 | 0.016 |
| K31 | R | R | R | R | R | R | R | R | R | R | R | R | R | R | S | 0.5 | ≤0.06 | 0.75 | 4 |
| K32 | R | R | R | R | R | R | R | R | R | R | R | R | R | R | S | 4 | 0.5 | 0.38 | 0.5 |
| K33 | R | R | R | R | R | R | R | R | R | R | R | R | R | R | S | 1 | ≤0.06 | 0.5 | 0.75 |
| K34 | R | R | R | R | R | R | R | R | R | R | R | R | R | R | S | 0.5 | ≤0.06 | 0.5 | 0.25 |
| K35 | R | R | R | R | R | R | R | R | R | R | R | R | R | R | R | 16 | 4 | 1.5 | 2 |
| K36 | R | R | R | R | R | R | R | R | R | R | R | R | R | R | S | 4 | 0.5 | 0.25 | 0.5 |
| K37 | R | R | R | R | R | R | R | R | R | R | R | R | R | R | S | 2 | 0.125 | 0.5 | 0.75 |
| K38 | R | R | R | R | R | R | R | R | R | R | R | R | R | R | S | 4 | 2 | 0.25 | 0.75 |
| K39 | R | R | R | R | R | R | R | R | R | R | R | R | R | R | S | 4 | 0.5 | 0.125 | 0.032 |
| K40 | R | R | R | R | I | I | R | R | R | R | R | R | I | I | S | 0.125 | ≤0.06 | 0.25 | 0.032 |
| K41 | R | R | R | R | R | R | R | R | R | R | R | R | R | R | S | 0.25 | ≤0.06 | 0.75 | 0.75 |
| K42 | R | R | R | R | R | R | R | R | R | R | R | R | R | R | S | 0.25 | ≤0.06 | 0.5 | 0.75 |
| K43 | R | R | R | R | I | I | R | R | R | R | R | R | R | R | S | 4 | 0.5 | 0.38 | 0.047 |
| K44 | R | R | R | R | R | R | R | R | R | R | R | R | R | I | S | 0.125 | ≤0.06 | 0.19 | 0.064 |
| K45 | R | R | R | R | R | R | R | R | R | R | R | R | R | R | S | 0.25 | ≤0.06 | 0.5 | 0.75 |
| K46 | R | R | R | R | R | R | R | R | R | R | R | R | R | R | S | 4 | 2 | 0.38 | 0.38 |
| K47 | R | R | R | R | R | R | R | R | R | R | R | R | R | R | S | 0.25 | ≤0.06 | 0.5 | 3 |
| K48 | R | R | R | R | R | R | R | R | R | R | R | R | R | R | S | 0.5 | 0.125 | 0.19 | 0.047 |
| K49 | R | R | R | R | I | I | S | I | S | S | S | R | S | I | S | 1 | 0.125 | 0.094 | 0.023 |
| K50 | R | R | R | R | I | I | R | R | R | R | R | R | R | R | S | 1 | ≤0.06 | 0.25 | 0.047 |
| K51 | R | R | R | R | R | R | R | R | R | R | R | R | I | I | S | 0.5 | ≤0.06 | 0.19 | 0.032 |
| K52 | R | R | R | R | R | R | R | R | R | R | R | R | R | R | S | 8 | 4 | 1 | 1.5 |
| K53 | R | R | R | R | R | R | R | R | R | R | R | R | R | R | S | 0.5 | ≤0.06 | 0.38 | 0.047 |
| K54 | R | R | R | R | R | R | R | R | R | R | R | R | R | R | S | 0.25 | ≤0.06 | 0.125 | 0.032 |
| K55 | R | R | R | R | R | R | R | R | R | R | R | R | R | R | S | 2 | ≤0.06 | 0.25 | 0.38 |
| K56 | R | R | R | R | I | I | R | R | R | R | R | R | R | R | S | 4 | 2 | 0.75 | 2 |
| K57 | R | R | R | R | R | R | R | R | R | R | R | R | R | R | S | 2 | 0.25 | 0.5 | 0.19 |
| K58 | R | R | R | R | R | R | R | R | R | R | I | R | S | R | R | 1 | 0.5 | 0.38 | 2 |
| K59 | R | R | R | R | R | R | R | R | R | R | R | R | R | R | S | 4 | 2 | 0.38 | 0.75 |
| K60 | R | R | R | R | R | R | R | R | R | R | R | R | R | R | S | 1 | 0.125 | 0.75 | 6 |
| K61 | R | R | R | R | R | R | R | R | R | R | R | R | R | R | S | 0.5 | ≤0.06 | 0.25 | 0.25 |
| K62 | R | R | R | R | R | R | R | R | R | R | R | R | R | R | S | 4 | 2 | 1 | 1.5 |
| K63 | R | R | R | R | R | R | R | R | R | R | R | R | R | R | S | 4 | 2 | 0.38 | 1 |
| K64 | R | R | R | R | R | R | R | R | R | R | R | R | R | R | S | 0.5 | ≤0.06 | 0.5 | 0.047 |
| K65 | R | R | R | R | R | R | R | R | R | R | R | R | I | R | S | 0.5 | 0.125 | 0.25 | 0.032 |
| K66 | R | R | R | R | R | R | R | R | R | R | R | R | I | I | S | 0.5 | 0.125 | 0.25 | 0.047 |

**Table S1.** The phenotypic susceptibility profiles of the 66 KPC-KP strains.

AMX; amoxicillin, AMC; amoxicillin-clavulanic acid, PIP; piperacillin, PTZ, piperacillin-tazobactam, TEM; temocillin, FOX; cefoxitin, CTX; cefotaxime, CAZ; ceftazidime, FEP; cefepime, ATM; aztreonam, ETP; ertapenem, IPM; imipenem, MEM; meropenem, CZA; ceftazidime-avibactam, FDC; cefiderocol, FDCA, cefiderocol-avibactam, IPR; imipenem-relebactam, MEV; meropenem-vaborbactam.

| **Isolate** | **Virulence score** | **Yersiniabactin** | **YbST** | **Colibactin** | **CbST** | **Aerobactin** | **AbST** | **Salmochelin** | **SmST** | **RmpADC** | **RmST** | **rmpA2** | **wzi** | **K_locus** | **O_locus** |
| --- | --- | --- | --- | --- | --- | --- | --- | --- | --- | --- | --- | --- | --- | --- | --- |
| K1 | 0 | - | 0 | - | 0 | - | 0 | - | 0 | - | 0 | - | wzi88 | KL157 | O1/O2v1 |
| K2 | 1 | ybt 14; ICEKp5 | 151-1LV | - | 0 | - | 0 | - | 0 | - | 0 | - | wzi83 | KL23 | O1/O2v2 |
| K3 | 0 | - | 0 | - | 0 | - | 0 | - | 0 | - | 0 | - | wzi154 | KL107 | O1/O2v2 |
| K4 | 0 | - | 0 | - | 0 | - | 0 | - | 0 | - | 0 | - | wzi321 | KL61 | O1/O2v1 |
| K5 | 0 | - | 0 | - | 0 | - | 0 | - | 0 | - | 0 | - | wzi187 | KL27 | O4 |
| K6 | 0 | - | 0 | - | 0 | - | 0 | - | 0 | - | 0 | - | wzi50 | KL15 | O4 |
| K7 | 1 | ybt 14; ICEKp5 | 151 | - | 0 | - | 0 | - | 0 | - | 0 | - | wzi83 | KL23 | O1/O2v2 |
| K8 | 0 | - | 0 | - | 0 | - | 0 | - | 0 | - | 0 | - | wzi154 | KL107 | O1/O2v2 |
| K9 | 0 | - | 0 | - | 0 | - | 0 | - | 0 | - | 0 | - | wzi177 | KL125 | O3/O3a |
| K10 | 0 | - | 0 | - | 0 | - | 0 | - | 0 | - | 0 | - | wzi168 | KL25 | O5 |
| K11 | 0 | - | 0 | - | 0 | - | 0 | - | 0 | - | 0 | - | wzi173 | KL102 | O1/O2v2 |
| K12 | 4 | ybt 14; ICEKp5 | 151 | - | 0 | iuc 1 | 1-2LV | - | 0 | - | 0 | - | wzi83 | KL23 | O1/O2v1 |
| K13 | 1 | ybt 9; ICEKp3 | 157-1LV | - | 0 | - | 0 | - | 0 | - | 0 | - | wzi173 | KL102 | O1/O2v2 |
| K14 | 1 | ybt 9; ICEKp3 | 202-1LV | - | 0 | - | 0 | - | 0 | - | 0 | - | wzi77 | KL57 | O1/O2v2 |
| K15 | 1 | ybt 16; ICEKp12 | 277-2LV | - | 0 | - | 0 | - | 0 | - | 0 | - | wzi173 | KL102 | O1/O2v2 |
| K16 | 0 | - | 0 | - | 0 | - | 0 | - | 0 | - | 0 | - | wzi173 | KL102 | O1/O2v2 |
| K17 | 1 | ybt 5; ICEKp6 | 10 | - | 0 | - | 0 | - | 0 | - | 0 | - | wzi37 | KL22 | O1/O2v1 |
| K18 | 1 | ybt 9; ICEKp3 | 183 | - | 0 | - | 0 | - | 0 | - | 0 | - | wzi137 | KL17 | O1/O2v1 |
| K19 | 1 | ybt 10; ICEKp4 | 28 | - | 0 | - | 0 | - | 0 | - | 0 | - | wzi154 | KL107 | O1/O2v2 |
| K20 | 1 | ybt 10; ICEKp4 | 28-1LV | - | 0 | - | 0 | - | 0 | - | 0 | - | wzi19 | KL19 | O1/O2v2 |
| K21 | 1 | ybt 13; ICEKp2 | 299-3LV | - | 0 | - | 0 | - | 0 | - | 0 | - | wzi29 | KL106 | O1/O2v2 |
| K22 | 1 | ybt 9; ICEKp3 | 157-1LV | - | 0 | - | 0 | - | 0 | - | 0 | - | wzi173 | KL102 | O1/O2v2 |
| K23 | 4 | ybt 9; ICEKp3 | 183 | - | 0 | iuc 1 | 1-1LV | - | 0 | - | 0 | rmpA2_6*-48% | wzi137 | KL17 | O1/O2v1 |
| K24 | 0 | - | 0 | - | 0 | - | 0 | - | 0 | - | 0 | - | wzi108 | KL118 | OL101 |
| K25 | 0 | - | 0 | - | 0 | - | 0 | - | 0 | - | 0 | - | wzi173 | KL102 | O1/O2v1 |
| K26 | 0 | - | 0 | - | 0 | - | 0 | - | 0 | - | 0 | - | wzi173 | KL102 | O1/O2v2 |
| K27 | 1 | ybt 14; ICEKp5 | 151 | - | 0 | - | 0 | - | 0 | - | 0 | - | wzi83 | KL23 | O1/O2v2 |
| K28 | 2 | ybt 17; ICEKp10 | 289-1LV | clb 3 | 17-1LV | - | 0 | - | 0 | - | 0 | - | wzi40 | KL3 | O1/O2v2 |
| K29 | 1 | ybt 9; ICEKp3 | 183-1LV | - | 0 | - | 0 | - | 0 | - | 0 | - | wzi137 | KL17 | O1/O2v1 |
| K30 | 1 | ybt 16; ICEKp12 | 277-2LV | - | 0 | - | 0 | - | 0 | - | 0 | - | - | KL102 | O1/O2v2 |
| K31 | 0 | - | 0 | - | 0 | - | 0 | - | 0 | - | 0 | - | wzi173 | KL102 | O1/O2v2 |
| K32 | 1 | ybt 13; ICEKp2 | 299 | - | 0 | - | 0 | - | 0 | - | 0 | - | wzi29 | KL106 | O1/O2v2 |
| K33 | 1 | ybt 13; ICEKp2 | 299 | - | 0 | - | 0 | - | 0 | - | 0 | - | wzi29 | KL106 | O1/O2v2 |
| K34 | 0 | - | 0 | - | 0 | - | 0 | - | 0 | - | 0 | - | wzi173 | KL102 | O1/O2v2 |
| K35 | 1 | ybt 9; ICEKp3 | 183 | - | 0 | - | 0 | - | 0 | - | 0 | - | wzi27 | KL27 | O1/O2v2 |
| K36 | 4 | ybt 14; ICEKp5 | 151-1LV | - | 0 | iuc 1 | 63 | - | 0 | - | 0 | rmpA2_6*-47% | wzi83 | KL23 | O1/O2v2 |
| K37 | 4 | ybt 9; ICEKp3 | 183 | - | 0 | iuc 1 | 1-1LV | - | 0 | - | 0 | rmpA2_6*-48% | wzi137 | KL17 | O1/O2v1 |
| K38 | 0 | - | 0 | - | 0 | - | 0 | - | 0 | - | 0 | - | wzi154 | KL107 | O1/O2v2 |
| K39 | 1 | ybt 10; ICEKp4 | 384-1LV | - | 0 | - | 0 | - | 0 | - | 0 | - | wzi19 | KL19 | O1/O2v2 |
| K40 | 2 | ybt 17; ICEKp10 | 289-1LV | clb 3 | 17-1LV | - | 0 | - | 0 | - | 0 | - | wzi40 | KL3 | O1/O2v2 |
| K41 | 0 | - | 0 | - | 0 | - | 0 | - | 0 | - | 0 | - | wzi173 | KL102 | O1/O2v2 |
| K42 | 0 | - | 0 | - | 0 | - | 0 | - | 0 | - | 0 | - | wzi173 | KL102 | O1/O2v2 |
| K43 | 0 | - | 0 | - | 0 | - | 0 | - | 0 | - | 0 | - | wzi27 | KL27 | O1/O2v2 |
| K44 | 1 | ybt 9; ICEKp3 | 183-2LV | - | 0 | - | 0 | - | 0 | - | 0 | - | wzi104 | KL51 | O1/O2v2 |
| K45 | 1 | ybt 9; ICEKp3 | 183-1LV | - | 0 | - | 0 | - | 0 | - | 0 | - | wzi173 | KL102 | O1/O2v2 |
| K46 | 1 | ybt 9; ICEKp3 | 183 | - | 0 | - | 0 | - | 0 | - | 0 | - | wzi137 | KL17 | O1/O2v1 |
| K47 | 0 | - | 0 | - | 0 | - | 0 | - | 0 | - | 0 | - | wzi173 | KL102 | O1/O2v2 |
| K48 | 0 | - | 0 | - | 0 | - | 0 | - | 0 | - | 0 | - | wzi82 | KL23 | O1/O2v2 |
| K49 | 0 | - | 0 | - | 0 | - | 0 | - | 0 | - | 0 | - | wzi168 | KL25 | O5 |
| K50 | 0 | - | 0 | - | 0 | - | 0 | - | 0 | - | 0 | - | wzi88 | KL150 | O3b |
| K51 | 2 | ybt 17; ICEKp10 | 289 | clb 3 | 17-1LV | - | 0 | - | 0 | - | 0 | - | wzi40 | KL3 | O1/O2v2 |
| K52 | 1 | ybt 9; ICEKp3 | 183 | - | 0 | - | 0 | - | 0 | - | 0 | - | wzi27 | KL27 | O1/O2v2 |
| K53 | 0 | - | 0 | - | 0 | - | 0 | - | 0 | - | 0 | - | wzi113 | KL111 | O3b |
| K54 | 1 | ybt 9; ICEKp3 | 183-2LV | - | 0 | - | 0 | - | 0 | - | 0 | - | wzi104 | KL51 | O1/O2v2 |
| K55 | 1 | ybt 13; ICEKp2 | 299 | - | 0 | - | 0 | - | 0 | - | 0 | - | wzi29 | KL106 | O1/O2v2 |
| K56 | 1 | ybt 9; ICEKp3 | 183-1LV | - | 0 | - | 0 | - | 0 | - | 0 | - | wzi137 | KL17 | O1/O2v1 |
| K57 | 1 | ybt 14; ICEKp12 | 561-1LV | - | 0 | - | 0 | - | 0 | - | 0 | - | wzi89 | KL110 | O1/O2v1 |
| K58 | 1 | ybt 9; ICEKp3 | 183-1LV | - | 0 | - | 0 | - | 0 | - | 0 | - | wzi173 | KL102 | O1/O2v2 |
| K59 | 0 | - | 0 | - | 0 | - | 0 | - | 0 | - | 0 | - | wzi154 | KL107 | O1/O2v2 |
| K60 | 0 | - | 0 | - | 0 | - | 0 | - | 0 | - | 0 | - | wzi154 | KL107 | O1/O2v2 |
| K61 | 1 | ybt 13; ICEKp2 | 299 | - | 0 | - | 0 | - | 0 | - | 0 | - | wzi29 | KL106 | O1/O2v2 |
| K62 | 1 | ybt 9; ICEKp3 | 157-1LV | - | 0 | - | 0 | - | 0 | - | 0 | - | wzi154 | KL107 | O1/O2v2 |
| K63 | 1 | ybt 9; ICEKp3 | 157-1LV | - | 0 | - | 0 | - | 0 | - | 0 | - | wzi154 | KL107 | O1/O2v2 |
| K64 | 1 | ybt 10; ICEKp4 | 384 | - | 0 | - | 0 | - | 0 | - | 0 | - | wzi173 | KL102 | O1/O2v2 |
| K65 | 0 | - | 0 | - | 0 | - | 0 | - | 0 | - | 0 | - | wzi177 | KL125 | O3/O3a |
| K66 | 1 | ybt 10; ICEKp4 | 89-2LV | - | 0 | - | 0 | - | 0 | - | 0 | - | wzi177 | KL114 | O1/O2v1 |

**Table S2.** Kleborate output of of the 66 KPC-KP strains.

| **Isolate** | **ST** | **KPC Variant** | **Tn4401 Isoform** | **Other Carbas/ESBLs** | **SHV Variant** | **Plasmid Replicons** |
| --- | --- | --- | --- | --- | --- | --- |
| K1 | 14 | KPC-3 | a |  | SHV-28 | FIIK, FIBpQIL, R, FIBK, FIB(pKPHS1), Col440I |
| K2 | 39 | KPC-2 | a | CTX-M-15, VEB-1 | SHV-11 | FIIK, FIBpQIL, C, ColRNAI |
| K3 | 512 | KPC-3 | a |  | SHV-11 | FIIK, FIBpQIL, FIB(NDM-Mar), HI1B(NDM-Mar), X3, ColRNAI |
| K4 | 517 | KPC-3 | b |  | SHV-11 | FIIK, FIA(HI1), Col440I |
| K5 | 392 | KPC-3 | a | CTX-M-15 | SHV-11 | FIIK, FIBpQIL, FIBK, ColRNAI |
| K6 | 37 | KPC-2 | a |  | SHV-11 | FIIK, FIBK, R, M1 |
| K7 | 39 | KPC-2 | a | VEB-1 | SHV-11 | FIBpQIL, FII(pKP91), C, ColRNAI, ColpVC |
| K8 | 512 | KPC-3 | a |  | SHV-11 | FIIK, FIBpQIL, FIBK, X3, ColRNAI |
| K9 | 2442 | KPC-3 | a | CTX-M-15 | SHV-77 | FIIK, FIBpQIL, FIA(pBK30683) |
| K10 | 258 | KPC-3 | b |  | SHV-11 | pKPC-CAV1193, R, ColRNAI |
| K11 | 307 | KPC-3 | a | CTX-M-15 | SHV-28 | FIBpQIL, FIBK |
| K12 | 39 | KPC-2 | a | CTX-M-15 | SHV-11 | FIIK, FIBpQIL, HI1B(NDM-Mar), ColRNAI |
| K13 | 307 | KPC-3 | a | CTX-M-15 | SHV-28 | FIIK, FIBpQIL, FIBK, ColBS512 |
| K14 | 23 | KPC-3 | a |  | SHV-1 | FIIK, FIBpQIL, FII, FII(pSDP9R), FIA(HI1), RepB, Col(CriePir75) |
| K15 | 307 | KPC-3 | a |  | SHV-28 | FIIK, FIBpQIL, FIB(pCAV1099-114), RepB |
| K16 | 307 | KPC-3 | a | NDM-1 | SHV-28 | FIIK, FIBpQIL, C, Col440I |
| K17 | 35 | KPC-3 | d |  | SHV-1 | FIBK, N |
| K18 | 101 | KPC-3 | a |  | SHV-1 | FIIK, FIA(HI1), R, ColRNAI, Col440II |
| K19 | 512 | KPC-3 | a |  | SHV-11 | FIIK, FIBK, X3 |
| K20 | 15 | KPC-3 | b | CTX-M-15 | SHV-28 | FIIK, FIBK, pKPC-CAV1193 |
| K21 | 258 | KPC-2 | a |  | SHV-11 | FIIK, FIBpQIL, FIBK, ColRNAI |
| K22 | 307 | KPC-3 | a | CTX-M-15 | SHV-28 | FIIK, FIBpQIL, FIBK |
| K23 | 101 | KPC-3 | a |  | SHV-1 | FIIK, FIBK, FIA(HI1), FIB(pNDM-Mar), HI1B(pNDM-Mar), R, ColRNAI, Col440II |
| K24 | 37 | KPC-39 | d | CTX-M-15 | SHV-11 | FIA(HI1), FIBK(pCAV1099-114), FII(pBK30683), R, repB |
| K25 | 45 | KPC-3 | a |  | SHV-1 | FIIK, FIBpQIL, FIBK |
| K26 | 307 | KPC-3 | a | CTX-M-15 | SHV-28 | FIIK, FIBpQIL, FIBK |
| K27 | 39 | KPC-2 | a | VIM-1 | SHV-11 | FIIK, FIBpQIL, FIB(AP001918), C |
| K28 | 13 | KPC-3 | d |  | SHV-1 | FII, FII(pBK30683), FIA(pBK30683), FIB(AP001918), Col156, ColRNAI |
| K29 | 101 | KPC-3 | a |  | SHV-1 | FIIK, FIA(HI1), R, ColRNAI, Col440II |
| K30 | 307 | KPC-3 | a | CTX-M-15 | SHV-28 | FIIK, FIBpQIL, FIB(pCAV1099-114), RepB |
| K31 | 307 | KPC-3 | a |  | SHV-28 | FIIK, FIBpQIL, FIBK, ColRNAI |
| K32 | 258 | KPC-2 | a | SHV-12 | SHV-12 | FIIK, FIBpQIL, FIBK, X3, ColRNAI |
| K33 | 258 | KPC-2 | a |  | SHV-1 | FIIK, FIBK, X3, RepB |
| K34 | 307 | KPC-3 | a |  | SHV-28 | FIIK, FIBpQIL, FIBK, ColRNAI |
| K35 | 661 | KPC-184 | a | CTX-M-15 | SHV-27 | FIIK, FIB(pCAZ1099-114), FIA(HI1), R, RepB, ColRNAI |
| K36 | 39 | KPC-2 | a | CTX-M-15 | SHV-11 | FIIK, FIBpQIL, FIB(pNDM-Mar), HI1B(pNDM-Mar), C, ColRNAI |
| K37 | 101 | KPC-3 | a |  | SHV-1 | FIIK, FIA(HI1), HI1B(pNDM-Mar), R, ColRNAI, Col440II |
| K38 | 512 | KPC-3 | a |  | SHV-11 | FIIK, FIBpQIL, FIBK, FIB(pKPHS1), X3, ColRNAI |
| K39 | 15 | KPC-3 | d | CTX-M-15 | SHV-28 | FIIK, FIBK, R, FIA(HI1) |
| K40 | 13 | KPC-3 | d |  | SHV-1 | FIA, FIA(pBK30683), FIB(AP001918), FII(pBK30683), FII(pRSB107), ColRNAI, Col156 |
| K41 | 307 | KPC-3 | a |  | SHV-28 | FIIK, FIBpQIL, FIBK, ColRNAI |
| K42 | 307 | KPC-3 | a |  | SHV-28 | FIIK, FIBpQIL, ColRNAI |
| K43 | 661 | KPC-3 | a | CTX-M-209 | SHV-27 | FIIK, FIBpQIL, FIB(pKPHS1), FIB(pCAV1099-114), RepB |
| K44 | 231 | KPC-3 | d |  | SHV-1 | FIIK, FIBK, FII(pBK30683), FIA(pBK30683) |
| K45 | 307 | KPC-3 | a |  | SHV-28 | FIBpQIL, C, ColRNAI |
| K46 | 101 | KPC-3 | a |  | SHV-1 | FIIK, FIBK, ColRNAI |
| K47 | 307 | KPC-3 | a |  | SHV-28 | FIIK, FIBpQIL, FIBK, ColRNAI |
| K48 | 280 | KPC-3 | d |  | SHV-27 | FIIK, FIBK, FIA(pBK30683), FII(pBK30683) |
| K49 | 258 | KPC-3 | b |  | SHV-11 | pKPC-CAV1193, ColRNAI |
| K50 | 1940 | KPC-2 | a |  | SHV-1 | FIBK, R, X3 |
| K51 | 13 | KPC-3 | d | CTX-M-15 | SHV-1 | FIBK, FIA(pBK30683), FII(pBK30683), ColRNAI |
| K52 | 661 | KPC-184 | a | CTX-M-15 | SHV-27 | FIIK, FIA(HI1), FIB(pCAV1099-114), R, RepB, ColRNAI |
| K53 | 1777 | KPC-3 | a |  | SHV-11 | X3 |
| K54 | 231 | KPC-3 | d |  | SHV-1 | FIIK, FIBK, FIA(pBK30683), FII(pBK30683) |
| K55 | 258 | KPC-2 | a |  | SHV-11 | FIIK, FIBK, X3, ColRNAI |
| K56 | 101 | KPC-3 | a |  | SHV-1 | FIIK, FIBK, FIA(HI1), R, ColRNAI, Col440II |
| K57 | 716 | KPC-3 | d | CTX-M-15 | SHV-27 | FIIK, FIBK, FIA(HI1), N, Col440I |
| K58 | 307 | KPC-272 | a |  | SHV-28 | FIIK, FIBpQIL, ColRNAI |
| K59 | 512 | KPC-3 | a |  | SHV-11 | FIIK, FIBpQIL, FIBK, X3, ColRNAI |
| K60 | 512 | KPC-3 | a | CTX-M-15 | SHV-11 | FIBpQIL, FIBK, ColRNAI |
| K61 | 258 | KPC-2 | a |  | SHV-1 | FIIK, FIBK, X3, ColRNAI |
| K62 | 512 | KPC-3 | a |  | SHV-11 | FIIK, FIBpQIL, FIBK, X3, ColRNAI |
| K63 | 512 | KPC-3 | a |  | SHV-11 | FIIK, FIBpQIL, FIBK, X3, ColRNAI |
| K64 | 8640 | KPC-3 | a | CTX-M-15 | SHV-28 | FIIK, FIBpQIL, FIBK |
| K65 | 2442 | KPC-3 | a | CTX-M-15 | SHV-77 | FIIK, FIBpQIL , FIA(pBK30683) |
| K66 | 219 | KPC-2 | NTE | CTX-M-15 | SHV-1 | C, FIB(pCAV1099-114), RepB |

**Table S3.** Genetic characteristics of the 66 KPC-KP isolates.

NTE; non-Tn4401 isoform,.
